# Encapsulation of *Lactobacillus gasseri*: Characterization, Probiotic Survival, In Vitro Evaluation and Viability in Apple Juice

**DOI:** 10.3390/foods11050740

**Published:** 2022-03-02

**Authors:** Abigail Varela-Pérez, Oscar O. Romero-Chapol, Ana G. Castillo-Olmos, Hugo S. García, Mirna L. Suárez-Quiroz, Jaspreet Singh, Claudia Y. Figueroa-Hernández, Rubí Viveros-Contreras, Cynthia Cano-Sarmiento

**Affiliations:** 1 Tecnológico Nacional de México/Instituto Tecnológico de Veracruz, Unidad de Investigación y Desarrollo en Alimentos, M.A. de Quevedo 2779, Veracruz 91897, Mexico; abigail_2365@hotmail.com (A.V.-P.); oscarosiel.rocha@outlook.com (O.O.R.-C.); anagpe_ibq@outlook.com (A.G.C.-O.); hugo.gg@veracruz.tecnm.mx (H.S.G.); mirna.sq@veracruz.tecnm.mx (M.L.S.-Q.); 2 School of Food and Advanced Technology, Massey University, Private Bag 11222, Palmerston North 4442, New Zealand; j.x.singh@massey.ac.nz; 3 CONACYT—Tecnológico Nacional de México/Instituto Tecnológico de Veracruz, Unidad de Investigación y Desarrollo en Alimentos, M.A. de Quevedo 2779, Veracruz 91897, Mexico; claudia.fh@veracruz.tecnm.mx; 4 Centro de Investigaciones Biomédicas, Universidad Veracruzana, Dr. Luis Castelazo Ayala S/N, Xalapa 91190, Mexico

**Keywords:** probiotic, encapsulation, viability, *Lactobacillus gasseri*

## Abstract

The development of functional foods containing probiotic bacteria has become increasingly relevant to improve and maintain health. However, this is often limited to dairy food matrices given the complexity involved in maintaining a stable system together with high microbial viability in matrices such as juices. The objective of this study was to develop and characterize sodium alginate capsules loaded with *Lactobacillus gasseri* ATCC^®^ 19992 ™ (LG). Cell viability under in vitro gastrointestinal conditions and during storage in apple juice were evaluated. The capsules were prepared by ionic gelation and an emulsification process was performed as pretreatment using two homogenization methods: magnetic stirring (AM) and Ultraturrax^®^ rotor-stator homogenizer (UT). Cell viability after encapsulation was similar in the two processes: 65%. At the end of the in vitro gastrointestinal evaluation, the non-encapsulated probiotic cells did not show any viability, while the AM system was able to retain 100% of its viability and the UT retained 79.14%. The morphology of the capsules consisted of a continuous and homogeneous surface. Cell viability of LG encapsulated in apple juice stored at 4 °C for 21 days was 77% for AM, 55.43% for UT, and 63.10% for free LG.

## 1. Introduction

A functional food can be defined as one that provides beneficial effects to the human body, in addition to its basic nutritional properties. Because of these properties, functional foods have gained importance and sometimes are part of the daily diet, which has prompted increased research and development efforts in this field [1,2,3]. Some of the favorable health effects of these foods have been related to osteoporosis, colon cancer, obesity, diabetes, constipation, and reduction of intestinal infections, among others [3]. The range of formulations and designs of these foods is broad and comprises from bioactive compounds to microorganisms. One of the latter functional components that have attracted attention are lactic acid bacteria (LAB) [4], due to their probiotic role. The International Scientific Association for Probiotics and Prebiotics (ISAPP) defines probiotics as “*live microorganisms that, when administered in adequate amounts, confer a health benefit on the host*” [5]. For probiotics to have a beneficial effect after administration, they must be in a concentration of 9–11 log CFU/day [6]; however, it is generally accepted that probiotic products should have a 6-8 log CFU/g [7]. Hence, such concentration is the one applied in functional products.

LAB are Gram-positive bacteria, characterized by having cocci or bacilli shapes. They are non-sporulating, acid-tolerant and produce lactic acid [8,9]. Within the LAB, the genera *Lactobacillus, Lactiplantibacillus, Lacticaseibacillus* and *Limosibacillus* contain several species classified as potentially probiotic microorganisms that have been employed in various clinical treatments. These include *Lacticaseibacillus rhamnosus* GG, *Lactiplantibacillus plantarum* 299, *Limosilactobacillus reuteri*, *Lacticaseibacillus casei*, *Lacticaseibacillus casei* LA1, *Lactobacillus gasseri,* among others [10].

*Lactobacillus gasseri* (LG) is a homofermentative, acid-resistant, and bile-tolerant native and indigenous human intestinal LAB [11,12]. It is frequently associated with probiotic benefits since it adheres to intestinal tissues, stimulates macrophages, and produces bacteriocins with the ability to reduce pathogenic microorganisms [13,14]. *L. gasseri* can degrade oxaloacetate in the gastrointestinal tract and maintain serum and renal homeostasis [14]. Also, its ability to protect the intestinal barrier has been reported, avoiding the entry of inflammatory substances as lipopolysaccharides from dietary intake with high-fat content to the circulation and therefore reduce the inflammation of adipose tissue [15].

Given the technological stress during its processing and physiological stress during its passage through the gastrointestinal tract to which the bacteria are subjected, their protection becomes relevant. This can be achieved through encapsulation to ensure their beneficial effects after consumption. Encapsulation is defined as the process where a compound or microorganism is trapped within a suitable matrix, affording systems with different particle sizes depending on the technique and energy used for their preparation [16]. There are several encapsulation techniques such as spray drying, freeze drying, fluidized bed drying, and ionic gelation. Ionic gelation was employed in the present work after performing an emulsification process as a pretreatment. This technique was selected because the physical crosslinking provided by ionic gelation is simple, fast, and inexpensive [17]. Encapsulation by ionic gelation allows bacteria to be immobilized within a matrix that provides a suitable environment as well as protection [18]; performing a prior emulsification involves less stress and damage to the bacteria, and good cell retention is achieved without using expensive reagents [19,20]; furthermore, another advantage of emulsification is the possibility to produce smaller capsules [21]. Calabuig-Jiménez et al. [22], Ding and Shah [23], and Patrignani et al. [24] used emulsification or homogenization processes prior to the formation of the capsules. Combining techniques can increase the viability of microorganisms, adequately control their release and improve the morphology of capsules loaded with lactic acid bacteria.

Probiotics are often added to dairy food matrices since they are their native growth media, but many people cannot tolerate dairy products. Thus, the development of alternative food matrices is relevant. Foods such as fruit juices have not been extensively explored in this context. A sufficiently stable system is required, which must be capable of protecting bacteria from low pH values (3–4.5) such as those found in fruit juices, because they could affect growth and stability of the LAB [25]. Despite their unusual utilization as probiotics carriers, juices provide a suitable environment for bacteria without severely changing their sensory properties, while retaining high microbial viability. For all the above reasons, the objective in this study was to prepare and characterize capsules loaded with *Lactobacillus gasseri* ATCC 19992 ™ (LG), and to evaluate their ability to survive under in vitro gastrointestinal conditions and during storage in apple juice. The capsules were prepared by ionic gelling using sodium alginate (ALG) as the encapsulating matrix that was subjected to an emulsification pretreatment.

## 2. Materials and Methods

### 2.1. Chemicals and Bacterial Strain

Freeze-dried granules of *Lactobacillus gasseri* ATCC 19992 ™ were acquired from the American Type Culture Collection (ATCC, Manassas, VA, USA). Sodium alginate, Tween^®^ 80, calcium chloride, de Man Rogosa and Sharp Medium (MRS), MRS agar, Gram stain kit, and all the reagents used to perform the in vitro evaluation were purchased from Sigma-Aldrich^®^ (St Louis, MO, USA). The commercial vegetable oil used in this study was bought from a local market in Veracruz (Mexico).

### 2.2. Activation and Preparation of Bacterial Culture

Freeze-dried *Lactobacillus gasseri* (LG) cells were rehydrated in 100 mL of sterile MRS broth (All-American 50X, Hillsville, VA, USA) and conditioned with CO_2_ for 3 min. Incubation was done at 37 °C for 24 h using a MaxQ Mini 4450 Shaker™ (Thermo Fisher Scientific^®^, Asheville, NC, USA). *Lactobacillus gasseri* was sub-cultured five times before being used in the study [26], and the recommendations of the strain supplier (ATCC) were also considered.

### 2.3. Preparation of Capsules Loaded with Lactobacillus gasseri

The bacterial culture was produced following the procedure of Patrignani et al. [24] with some modifications. In brief, an alginate mixture (ALG) containing 100 mL of 3% sodium alginate (*w*/*v*) was combined with 25 mL of bacterial suspension with a *Lactobacillus gasseri* concentration of approximately 7.1 log CFU/mL. Subsequently, 1 mL of Tween^®^ 80 and 100 mL of vegetable oil were added. The system was subjected to an emulsification process as a pretreatment following the method of Ding and Shah [23] with modifications: using either a magnetic stirrer (AM) (PC-420D, Corning^®^, NY, USA) set at 1150 rpm for 10 min, or an Ultraturrax^®^ (UT) digital T-25 rotor-stator homogenizer (IKA^®^, Staufen, Germany) at 4000 rpm for 7.5 min. After the pretreatment, the system was dispersed in a 0.1 M calcium chloride solution by dripping with a 21G needle placed 15 cm above the solution with a flow of 2.5 mL/min using an RD100-01 peristaltic pump (Ecoshell^®^ Pharr, TX, USA) fitted with a YZ1515 head and a silicone hose # 19 (Qilipump, Hebei, China) forming the capsules with *L. gasseri*. Finally, once the capsules were shaped, they were stored in a 0.1 M calcium chloride solution for 12 h at 4 °C to promote further crosslinking [23,27].

### 2.4. Lactobacillus gasseri Viability 

Viability and encapsulation efficiency of the bacteria were evaluated by dissolving 1 g of capsules in PBS solution (pH 7) for 15 min with shaking at 2000 rpm with a vortexer (Genie-2, Scientific Industries Inc., Bohemia, NY, USA); 100 μL were used as inoculum for subsequent serial dilutions and colonies were counted. In the case of free cells, the buffer and stirring were omitted. For the viability evaluation, Equation (1) was used:

(1) $$EE\;\left(\%\right)\;=\;\frac{\log N}{\log N_{0}}\;\times \;\left(100\right)$$

Viability is given in percentage, *N_0_* is the number of bacteria (log CFU/mL) before encapsulation, and *N* is the number of bacteria (log CFU/mL) released by the capsules [23,28,29,30].

### 2.5. Morphological Analysis of Free and Encapsulated Lactobacillus gasseri

The morphology and microstructure of the capsules, as well as the free bacteria, were analyzed using scanning electron microscopy (SEM) following the method of Silva et al. [31] with some modifications concerning the voltage used and the drying treatment that the capsules received before being observed by SEM. The equipment used was a MIRA3 system (Tescan^®^, Brno, Czech Republic). The capsules were dried for 1 h in a laminar flow biosafety cabinet (A2 1300 Series, Thermo Fisher Scientific^®^, Asheville, NC, USA) at 25 °C and subsequently placed on carbon tape. The conditions were set with a voltage of 10 kV, with a low vacuum mode of operation, in addition to using a secondary electron detector (SE) at a working distance of 10 mm. The images obtained by the scanning electron microscope were processed with the Mira3 software version 4.2.19.1 (Tescan^®^, Brno, Czech Republic) in TIFF format.

The morphology and microstructure of the capsules, as well as the free bacteria, were analyzed using scanning electron microscopy (SEM) following the method of Silva et al. [31] with some modifications concerning the voltage used and the drying treatment that the capsules received before being observed by SEM. The equipment used was a MIRA3 system (Tescan^®^, Brno, Czech Republic). The capsules were dried for 1 h in a laminar flow biosafety cabinet (A2 1300 Series, Thermo Fisher Scientific^®^, Asheville, NC, USA) at 25 °C and subsequently placed on carbon tape. The conditions were set with a voltage of 10 kV, with a low vacuum mode of operation, in addition to using a secondary electron detector (SE) at a working distance of 10 mm. The images obtained by the scanning electron microscope were processed with the Mira3 software version 4.2.19.1 (Tescan^®^) in TIFF format.

Morphometric parameters such as Feret diameters, area, and circularity were obtained by 24.5 megapixel photographs in JPG format taken with a D3200 camera (Nikon^®^, Minato, Tokyo, Japan) placed 15 cm away from the target. For this analysis, approximately 100 capsules were placed in a Petri dish with a millimeter paper at the bottom and the ImageJ 1.52q software was used for the analysis [32]. The circularity of the capsules was calculated with Equation (2) [33]:

(2) $$Circularity=\frac{4\pi A}{P^{2}}$$

where *A* is the area of the capsule and *p* is the perimeter. If the capsules produce values close to 1, they are interpreted as a morphology that geometrically resembles a perfect circle.

To visualize free cells of *Lactobacillus gasseri*, a DM2000 LED optical microscope (Leica Microsystems^®^, Wetzlar, Germany) and the Leica Application Suite V4.9 software were used at 100x magnification, performing Gram staining. LG cells were visualized using SEM; the cells were dried for 8 h on carbon tape in a laminar flow biosafety cabinet at 25 °C. The conditions were set with a voltage of 10 kV, with a low vacuum mode of operation, in addition to using a secondary electron detector (SE) at a working distance of 10.98 mm.

### 2.6. Viability of Lactobacillus gasseri Encapsulated in Juice

For this analysis, the capsules loaded with LG and free cells were added to the food matrix; both samples were stored at 4 °C for three weeks. The product used was Jumex Único Fresco^®^ apple juice. The juice was treated with CO_2_ to generate anaerobic conditions. The viability of LG was monitored as described above by equation 1:1 g of capsules taken and dissolved in PBS (pH 7). In the case of free bacteria, 100 μL of juice with LG were taken for the culture. Serial dilutions and counting were carried out as described above [34,35].

### 2.7. Viability of Free and Encapsulated Lactobacillus gasseri in Simulated Gastrointestinal Conditions 

For gastrointestinal simulation, the COST INFOGEST in vitro digestion method was followed [36,37]. For the oral phase, 5 g of capsules were taken from each system or 5 mL of free cells. Later, they were mixed with 3.5 mL of salivary solution that consisted of: 0.5 mL of α-amylase solution at 1500 U/mL, 25 µL of 0.3 M CaCl_2_, and 975 µL of distilled water and incubated for 2 min at 37 °C; then 7.5 mL of previously prepared gastric electrolyte stock solution for the gastric phase that consisted of 1.6 mL of pepsin solution of 25,000 U/mL, and 5 μL of 0.3 M CaCl_2_ were added. The pH of the solution was adjusted to 3 ± 0.2 with 1 M HCl and made up to 20 mL with distilled water. The gastric phase was incubated at 37 °C for 2 h. At the end of the gastric phase, the samples were cooled in an ice bath before the intestinal phase was prepared. For the last stage, 20 mL from the gastric phase was mixed with 11 mL of intestinal electrolyte stock solution, 5 mL of pancreatin at 100 U/mL, 40 μL of 0.3 M CaCl_2_, and 2.5 mL of 9% bile solution. The pH value was adjusted to 7.0 ± 0.2 with 1 M NaOH and made up to 40 mL with distilled water; the phase was later incubated at 37 °C for 2 h at 95 rpm. Incubations of all solutions were carried out in the orbital Shaker. At the end of each stage of the digestion, aliquots of both encapsulated and free LG systems were taken to perform viability analysis by plating on a Petri dish and Equation (1) and the visual inspection in SEM under the previously described conditions.

### 2.8. Statistical Analysis

Statistical analysis was performed by analysis of variance (ANOVA) and comparing means by Tukey’s test using MiniTab v. 19.1 software (Minitab Inc., State College, PA, USA). The level of significance was set at *p* ≤ 0.05. The experiments were performed in duplicate and the data represent the means ± standard deviation.

## 3. Results and Discussion

### 3.1. Viability of Capsules Loaded with Lactobacillus gasseri

After 24 h of incubation, a 7.1 log CFU/mL concentration was obtained for *Lactobacillus gasseri* before encapsulation. This concentration was considered suitable, according to Kechagia et al. [38], who suggest that concentrations above 6 log CFU/mL are generally accepted as beneficial. With that reference, the encapsulation process was performed with its respective pretreatment.

The viability of encapsulated and free LG was determined. It showed values of 65.73% for the magnetic stirring pretreatment and 65.90% for the UT pretreatment (Table S1). Ding and Shah [23] performed tests where they encapsulated various LAB with different pretreatments, AM and UT, and found that smaller capsules were produced when long times were used, coupled to higher speeds (rpm). However, lower viabilities were also obtained, specifically for treatments of 10 min at 4000 rpm and 5 min at 8000 rpm. The authors obtained losses in viability in approximately 2.93 log CFU/mL. A similar reduction was reported in this work since a decline in viability of approximately 1.762 log CFU/mL was measured, which indicates that a time of 7.5 min with 4000 rpm produced better viabilities. Comparing the work of Ding and Shah [23] and this study, the composition of constituents is similar. However, the amount of oil used is significantly lower in this study; while they used 800 mL of vegetable oil, in the present work, only 100 mL were employed. The emulsification process in this study was made following the method of Ding and Shah [23] with some modifications; the concentration of oil was selected based on preliminary studies (data not shown), where capsules with fragile and irregular morphology and larger sizes were obtained. Furthermore, phase separation developed, a phenomenon indicated as a disadvantage when preparing an emulsion [21]. With modifications in the methodology, capsules with regular and homogeneous morphology were obtained, which were more rigid and smaller in size. With this composition, it is suggested that the addition of oil to the encapsulating matrix improves the morphology of the capsules in addition to enhance cell viability possibly through the presence of fatty acids in the vegetable oil and in Tween that increase cell survival under stress conditions [39,40], compared to the systems reported in the literature of encapsulation in a mixture of alginate alone [30,41], so the use of an emulsion suggests an advantage in the encapsulation of bacteria.

On the other hand, Chávarri et al. [30] carried out encapsulation studies on *L. gasseri*, where a layer of chitosan was added to the alginate capsules loaded with LAB and obtained a 39.2 ± 2.3% encapsulation yield. This yield was smaller than the value we obtained in this work with the two different pretreatment methods (AM and UT), despite not having subjected the capsules to any coating. It is known that the encapsulation efficiency is influenced and depends on various factors such as the alginate concentration (wall material), the concentration and species of the probiotic cells, the content of calcium chloride, and the hardening time after encapsulation [42,43].

Ding and Shah [27] reported high viability values after encapsulation with a concentration of 3% (*w*/*v*) of alginate with various species of LAB. Therefore, it was considered as the optimal concentration to encapsulate *L. gasseri*. In addition to the alginate concentration, the 12 h hardening time can lead to elevated viability. The losses in viability can be attributed to sensitivity of LG to shear stress.

### 3.2. Morphological Analysis of Lactobacillus gasseri

**Figure 1:**
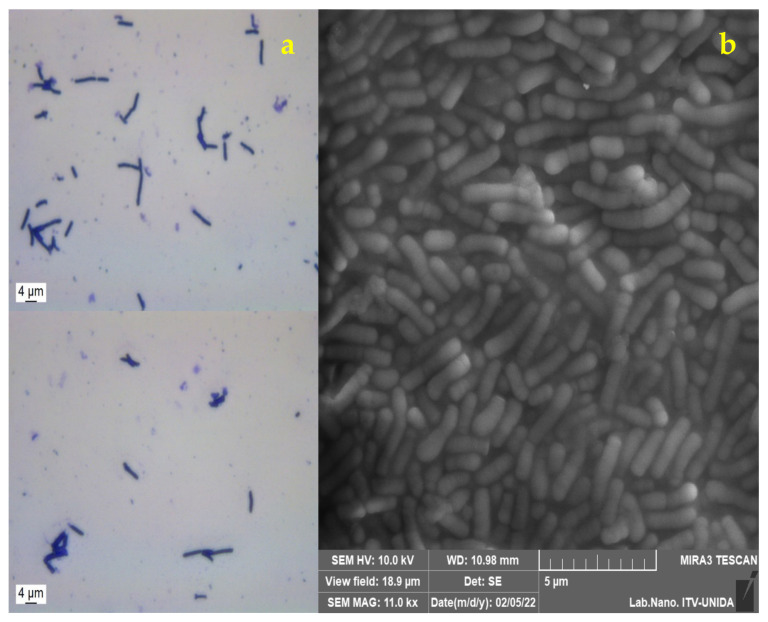
Micrographs of *Lactobacillus gasseri*: (**a**) optical microscopy (100×); (**b**) scanning electron microscopy (SEM).

Gram staining was applied to *L. gasseri*, which was then visualized by optical microscopy (Figure 1a) [12]. In addition, the morphology was analyzed by SEM, where its elongated bacillary shape, with rounded ends and sizes between 4.48 and 5 μm was verified. (Figure 1b) which corresponds to the report by Azcarate-Peril et al. [44], who found that the measurements corresponding to *L. gasseri* were 0.6 to 0.8 by 3 to 5 μm.

The morphology of the capsules obtained by the dripping technique with the two pretreatments was different from each other. The pretreatment with UT was the one with the best circularity value, followed by AM. Since these values are close to 1, it is inferred that the capsules are mainly regular and spherical. Maximum and minimum Feret diameter data are shown in Table 1. These results were obtained with the ImageJ software from the pictures obtained from the capsules with *L. gasseri*. The measurements of the capsules obtained in this study were similar to those reported by Muthukumarasamy et al. [45], where the use of the extrusion technique to encapsulate *Lactobacillus reuteri* using alginate as wall material, produced capsules with a mean diameter of 2.37 mm.

In the present work, a mean diameter of 2.599 ± 0.122 mm was measured for AM and 2.915 ± 0.196 mm for UT (Table 1). The size values of the capsules varies depending on the encapsulation technique used. In the case of extrusion, the size depends on the dimensions of the needle. At the same time, the factors that can influence the size of capsules produced from emulsification are the stirring speed, and the type of emulsifier used [18]. In the study, different sizes were obtained in the capsules even when the same encapsulation technique and emulsifier were employed. These data could have been obtained because two different pretreatments with different rpm were used, so the different sizes are attributed to the applied stirring speed and the AM and UT homogenization process. During the homogenization process to obtain the emulsion, AM and UT generate disruptive forces that interrupt the interface between oil and water, in such a way that droplet sizes are reduced, which affects the final size of the capsules [23].

**Figure 2:**
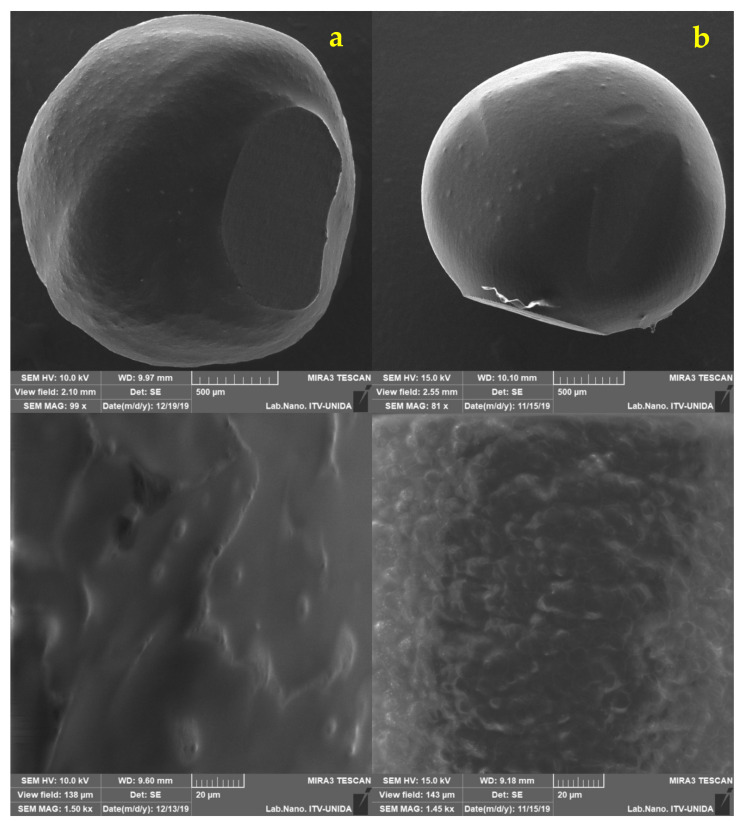
Micrographs from scanning electron microscopy of the LG alginate (ALG) systems made by ALG AM magnetic stirring (column (**a**)), rotor-stator ALG UT (column (**b**)) drip homogenization.

The size and shape of the capsules were analyzed by SEM (Figure 2). In the micrographs, a regular morphology resembling a sphere is confirmed. A similar morphology was described by Pourjafar et al. [46], who prepared capsules loaded with *L. acidophilus* and *L. rhamnosus* with either a double coating (chitosan and Eu S100) or a single coating (chitosan). The authors obtained a smooth morphology on the surface of the capsules with double coating, while for the capsules with a single coating they reported an irregular and rough appearance. These results are similar to those obtained in this study since capsules with slightly rough homogeneous surfaces were produced, despite not receiving a coating. It has been shown that if the capsules have a smooth surface, they display better resistance to adverse conditions, while a rough and porous surface is generally weaker [46].

No fractures were observed on the surface of capsules obtained with the two pretreatments (AM and UT). The shape of *L. gasseri* cells can be observed on the surface, not in a fully exposed way, but it is clearly appreciated that the bacteria are distributed. This type of conformation can be seen in the micrographs previously reported by Jiménez-Pranteda et al. [47], in which the capsule formed with xanthan:gellan gum is shown and how the *Lactobacillus* cells are homogeneously distributed within the matrix forming layers from the core to the surface.

### 3.3. Viability of Encapsulated Lactobacillus gasseri in Apple Juice

Once the juice samples were inoculated with encapsulated bacteria and free LG, they were monitored for three weeks. Each week, 1 g of capsules were withdrawn, and their viability was evaluated; thus, the number of viable cells was quantified for the two systems in apple juice kept at 4 °C. In this way, the effect this food matrix represented for the structure of the capsules was obtained (Table 2).

LG had a final viability of 77% (4.497 log CFU/mL) for AM and 55% (2.304 log CFU/mL) for the UT treatment. The free bacteria had a viability of 63%, suggesting that the capsules produced by AM and UT provided LG with a suitable environment for its survival against the acidic conditions of apple juice since the capsules with alginate very likely provided an anaerobic environment [20]. Rodrigues et al. [48] reported that when encapsulating *L. paracasei* L26 in alginate with chitosan and dextran sulfate, there was no evidence of any beneficial effect of the capsule coating; this highlights the ability of alginate to provide a suitable environment for bacteria in food matrices such as juices.

On the other hand, free LG showed a decrease in viability of 6.7 log CFU/mL to 4.232 in the last week. The decrease in viable cells of free LG can be related to the use of carbohydrates present in the juice. While consuming carbohydrates, bacteria release organic acids further increasing the acidify of the juice [34], producing a more hostile environment that could cause a decrease in cell viability as observed during the final week of the study. The decrease of viable probiotic cells in the juice has also been reported by Shah et al. [49], who reported a decrease in the viability of strains such as *L. rhamnosus*, *Bifidobacterium lactis*, and *L. paracasei* L26 over time in model fruit juices.

**Figure 3:**
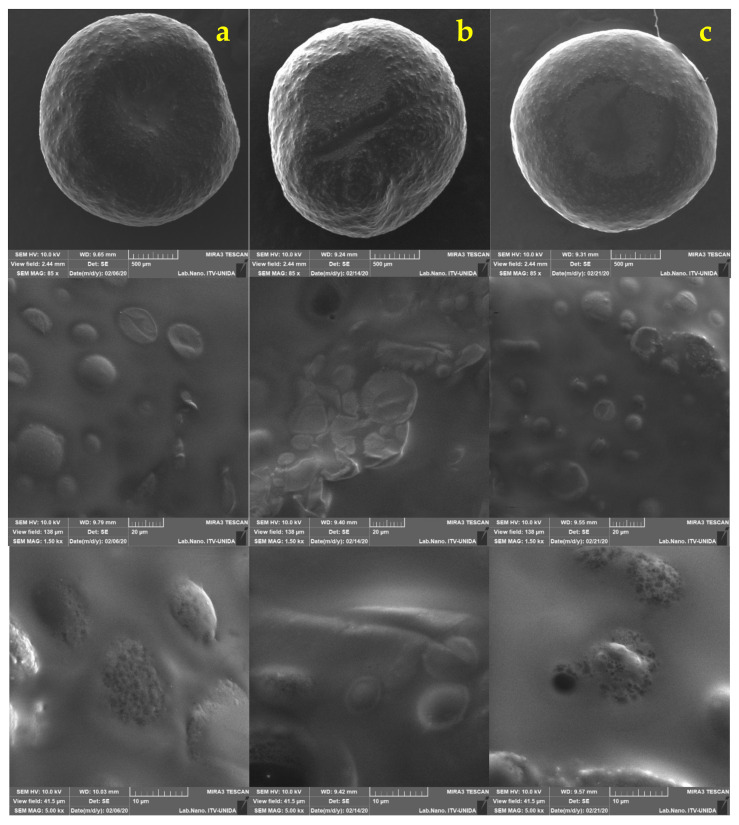
*Lactobacillus gasseri* (LG) encapsulated in apple juice with CO_2_ by ALG AM: (column (**a**)) 7 days, (column (**b**)) 15 days, and (column (**c**)) 21 days in refrigerated storage (4 °C).

**Figure 4:**
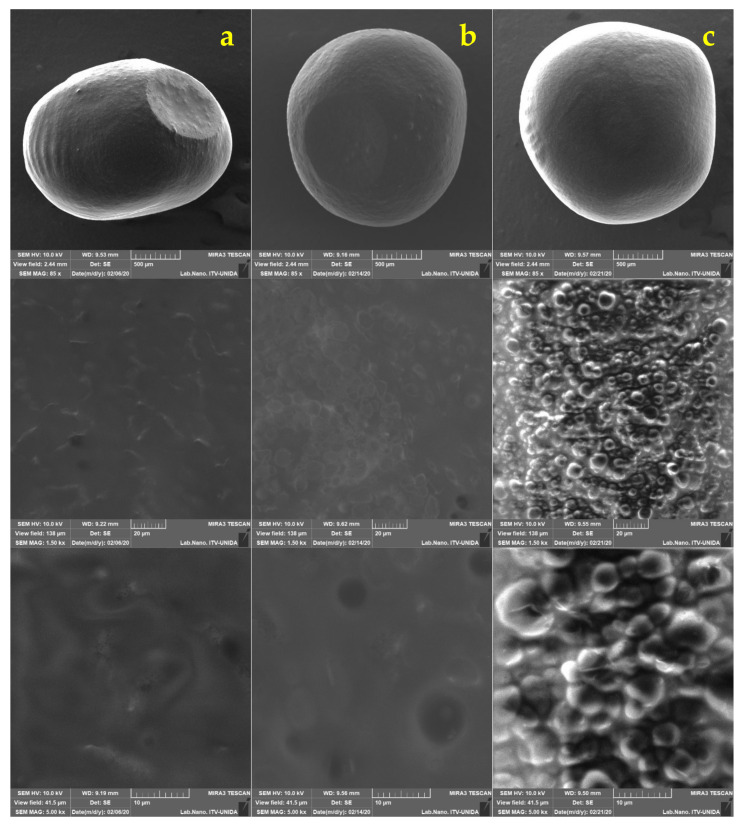
*Lactobacillus gasseri* (LG) encapsulated in apple juice with CO_2_ by ALG UT: (column (**a**)) 7 days, (column (**b**)) 15 days, and (column (**c**)) 22 days in refrigerated storage (4 °C).

The decline and upsurge of viable cells in the capsules may be attributed to exposure to low pH values for rather long periods [35]. Further analysis by SEM were performed on the capsules every week to examine if surface changes occurred (Figure 3 and Figure 4). Minimal differences in morphology were found during the three weeks of refrigerated storage. Humidity is an important aspect when inspecting samples in SEM. In this work, it was observed that the longer the capsules remained in the juice, the more moisture they contained. This behavior made the analysis difficult from the second week since the electron beam could have pierced the capsule until its rupture and, consequently, the components were released after long focus times.

### 3.4. Viability of Free and Encapsulated Lactobacillus gasseri under Simulated Gastrointestinal Conditions

Due to the wide variety of methodologies currently available designed to simulate the digestion process, comparing results becomes a complicated task. However, the same exposure times and similarities in phase composition or the COST INFOGEST technique were followed [36].

At the end of each simulation phase, aliquots were taken to quantify the number of cells released from the capsules. At the beginning of the gastrointestinal simulation, the capsules with LG had viability values of 4.961, 4.895 log CFU/mL for LG ALG AM and LG ALG UT treatments, respectively, and 7.09 log CFU/mL LG free cells. For this analysis, the oral phase (salivary) was optional because the capsules were designed to be incorporated in liquid foods, and therefore the salivary phase could be omitted [37]. However, as the behavior of free LG was evaluated, it was decided to carry it out, although a number of authors do not consider this approach.

Free LG cells in the salivary phase showed a reduction of at least two log cycles. For the gastric phase, the viability continued to drop; however, viable bacteria were still found. A similar trend was reported by Jurado-Gámez et al. [13], who exposed *L. gasseri* to pH values of 6, 4.5, and 3.5 for 3 h. At the end of their experiments, they reported viable cells, indicating that *L. gasseri* can tolerate and survive acidic environments in vitro. However, Chávarri et al. [30] also conducted studies on *L. gasseri*, and found that, after exposure to simulated gastric fluid (pH 2) for 2 h, the viability of LG free cells was 10 CFU/mL (detection limit). Those studies suggest that *L. gasseri* free cells can survive in simulated gastric conditions at pH values of 2 or 3.

After the bacteria survive the conditions of the gastric phase, they enter the intestinal phase, where bile is one of the main challenges, since it has a detergent-like properties with antimicrobial activity through dissolution of the bacterial membrane [50,51,52]. On the other hand, not only bile can affect bacterial viability, but also digestive enzymes can affect survival by damaging the cell membrane and DNA [53]. The behavior described is possible since the cell count was zero for the intestinal phase. A similar result was reported by Ortakci and Sert [41], who indicated that free *L. acidophilus* ATCC 4356 did not survive when subjected to a simulated intestinal phase.

**Figure 5:**
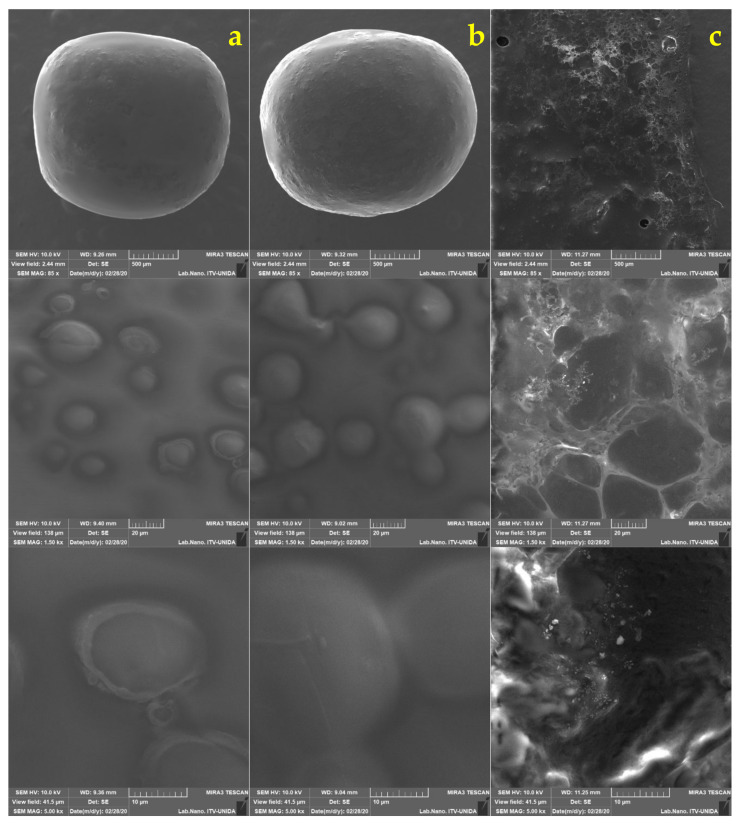
*Lactobacillus gasseri* encapsulated by ALG UT. Salivary phase (column (**a**)), gastric phase (column (**b**)), and intestinal phase (column (**c**)).

**Figure 6:**
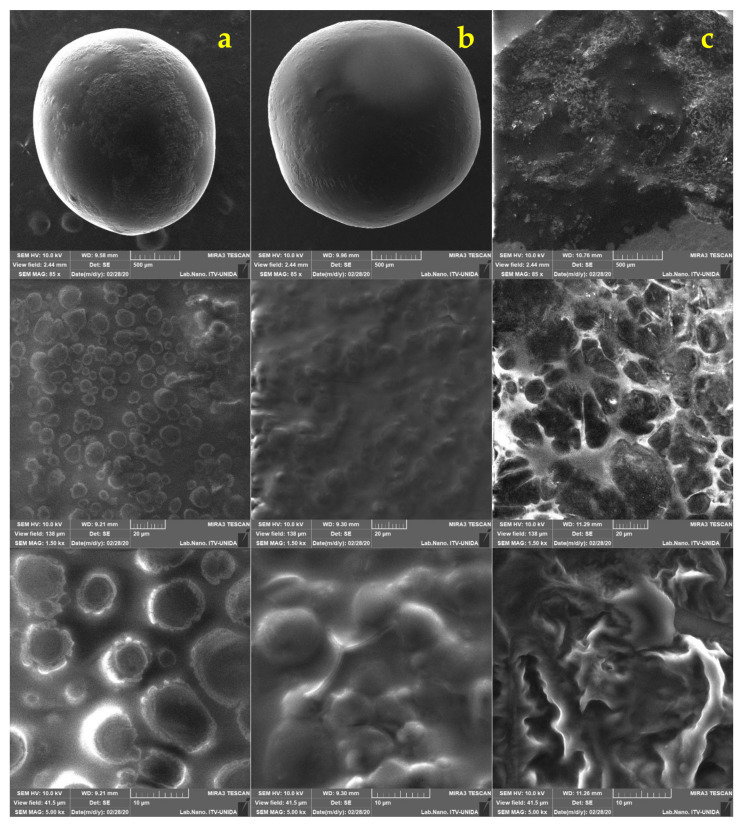
*Lactobacillus gasseri* encapsulated by ALG AM. Salivary phase (column (**a**)), gastric phase (column (**b**)), and intestinal phase (column (**c**)).

The capsules loaded with LG both ALG AM and ALG UT showed similar trends in the salivary phase (release of 2 log CFU/mL), while for the gastric phase ALG AM had a release of 3.77 log CFU/mL; that is, this treatment experienced more significant cell losses in the capsules compared to ALG UT, which preserved a release of 2 log CFU/mL. These viability losses can be attributed to exposure to low pH value (pH 3) [41,54]. Finally, in the intestinal phase, the capsules exhibited a rupture phenomenon (Figure 5c and Figure 6c). It is inferred that the alginate capsules provided the bacteria with an ideal environment to survive pancreatin and bile salts for the time necessary to avoid a viability loss due to the antimicrobial effect of bile salts [31]. It has been reported that alginate capsules change as they are subjected to different conditions. With gastric juice, the walls are compressed, and, in contrast, with intestinal juice, an expansion occurs [55]. Despite the above, the capsules remained unchanged in the gastric phase. It was not until the intestinal phase that they were dilated, which is consistent to the report by Silva et al. [56], where when encapsulating *Lactobacillus acidophilus* with ALG, they report the same behavior, and it was attributed to the instability of the particles when exposed to neutral pH; the phenomenon by which an expansion occurs can be explained since the ALG being in neutral pH, its carboxyl groups are exposed to bile salts, and with this, an ionic exchange occurs, resulting in the relaxation of the chains and therefore the expansion and eventual rupture [56].

Table 3 and Figure S1 show the viable cell count at the beginning and end of the gastrointestinal simulation. Free cells of *L. gasseri* had a higher number of CFUs. At the end of the simulation, it did not show any viability. However, LG UT had 79.14% and LG AM 100% viable cells, the latter being the better treatment. Zeashan et al. [57] obtained alginate capsules loaded with *Lactobacillus acidophilus*. When subjected to gastric and intestinal simulation, they obtained a viability of approximately 90% for both simulations, despite being different species of *Lactobacillus* and performing the gastric and intestinal phase separately. In this work, where the simulation was continuous, the highest viability was obtained with LG AM treatment.

The LG AM treatment can be compared with the results obtained by Chávarri et al. [30] when subjecting alginate capsules loaded with LG to intestinal conditions. They obtained a viability of 98.86%. It should be mentioned that their capsules were obtained by extrusion. Therefore, greater viability could be attributed to the encapsulation method and its pretreatment. When comparing the final viabilities of the gastrointestinal simulation, Lee et al. [58] reported viabilities of 41% for alginate capsules with *Lactobacillus acidophilus* KBL409 obtained by extrusion, which is a lower value than those obtained in this work with either treatment, reinforcing the idea that encapsulation by ionic gelling with sodium alginate and an emulsification process as a pretreatment is a more viable option to encapsulate *Lactobacillus* since it protects bacteria better, at least under simulated gastrointestinal conditions.

The micrographs in Figure 5 and Figure 6 show comparisons of the different stages of the simulation for each treatment, and the viability (%) data are verified since no significant changes were visually observed in the capsules in the salivary and gastric phases. Therefore, it can be assumed that alginate protects *L. gasseri* cells during these phases. At the end of the intestinal phase, the capsules did show noticeable texture changes since they broke at the slightest touch. In order to be able to visualize them with the microscope, a liquid sample of the medium that contained the capsule (already dissolved) was taken and subjected to the same conditions. The capsule-breaking phenomenon was previously reported by Chun et al. [17] who prepared capsules loaded with *L. plantarum* DKL109 by external ionic gelling with an atomizer spraying device and with wall materials made of 1.5% alginate in combination with 3% gum Arabic; these capsules when subjected to artificial gastric juice for 2 h at pH 2 and subsequently to artificial intestinal juice for 2 h at a pH of 7 with bile acid, and then visually inspected with light microscopy. The capsules retained their uniform spherical morphology during the gastric phase, while they ruptured during the intestinal phase.

## 4. Conclusions

The formulation of functional foods has attracted increasing interest over time, specifically foods that contain probiotic lactic acid bacteria because they can provide health benefits and represent a growing market in the food sector. The application of potentially probiotic LABs has been associated with a wide range of dairy products. However, they are currently being incorporated into different food matrices such as juices, despite the challenges of preserving microbial viability in these matrices. In this work, favorable results were obtained by encapsulating *L. gasseri* with ALG and carrying out an emulsification process as pretreatment (AM and UT). An encapsulation efficiency of approximately 65% was obtained for both pretreatments together with almost smooth spherical morphologies, and only slight roughness. One of the objectives to be met was the storage of capsules loaded with LG in apple juice, where viabilities of 77% for AM and 55.43% for UT were obtained, thus verifying that encapsulation with ALG provides bacteria with a suitable environment for their survival. Under simulated gastrointestinal conditions, free *L. gasseri* did not retain its viability (0%), which confirms that this bacterium requires encapsulation in order to survive this process. The capsules of both AM and UT remained undegraded during the salivary and gastric phases, thus helping to maintain the viability of 100 and 79% for ALG AM and ALG UT, respectively. These results indicate that the encapsulation method provided protection to *L. gasseri* under simulated gastric and intestinal conditions; however, even when high viabilities were obtained, the concentration used was not enough to meet the minimum necessary intake (6 log CFU/mL) that probiotic bacteria require to provide a beneficial effect to their host. Given the high sensitivity of the strain, it would be necessary to use high concentrations in the food matrices so that the minimum necessary population can reach the desired target.

## Figures and Tables

**Table 1:** Results of the digital analysis of images of the encapsulation methods for *Lactobacillus gasseri*.

Encapsulation Method	Maximum Feret Diameter (mm)	Minimum Feret Diameter (mm)	Circularity
ALG UT	3.284 ± 0.304 ^a^	2.915 ± 0.196 ^a^	0.885 ± 0.092 ^a^
ALG AM	2.856 ± 0.123 ^b^	2.599 ± 0.122 ^b^	0.854 ± 0.019 ^b^

ALG AM = Alginate with pretreatment magnetic stirring; ALG UT = Alginate with pretreatment Ultraturrax®. Means ± SD (*p* < 0.05). Equal letters in a row means that there is no statistically significant difference.

**Table 2:** Survival of *Lactobacillus gasseri* (LG) in juice from ALG AM, ALG UT, and cell-free systems in refrigerated storage (4 °C).

Method	log CFU/mL (N_0_)	log CFU/mL (N)	Viability (%)
ALG AM	5.771 ± 0.326 ^a^	4.497 ± 0.075 ^b^	77
ALG UT	4.151 ± 0.213 ^a^	2.304 ± 0.04 ^b^	55.43
Free LG	6.7 ± 0.023 ^a^	4.232 ± 0.089 ^b^	63.10

ALG AM = Alginate with pretreatment magnetic stirring; ALG UT = Alginate with pretreatment Ultraturrax^®^. N_0_ = initial viability; N= final viability. Means ± SD (*p* < 0.05). Equal letters in a column means that there is no statistically significant difference.

**Table 3:** Viability of *Lactobacillus gasseri* at in vitro gastrointestinal conditions of the ALG AM, ALG UT, and cell-free systems (*n* = 2).

Method	Initial log CFU/mL (N_0_)	Final log CFU/mL (N)	Viability (%)
ALG AM	4.961 ± 0.243 ^a^	4.962 ± 0.166 ^a^	100
ALG UT	4.895 ± 0.025 ^a^	3.875 ± 0.087 ^b^	79.14
Free LG	7.09 ± 0.014 ^a^	No growth ^b^	0

ALG AM = Alginate with pretreatment magnetic stirring; ALG UT = Alginate with pretreatment Ultraturrax^®^. N_0_ = initial viability; N= final viability. Means ± SD (*p* < 0.05). Equal letters in a column means that there is no statistically significant difference.

## Data Availability

The authors declare the transparency of data.

## Supplementary Materials

The following are available online at https://www.mdpi.com/2304-8158/11/5/740/s1, Figure S1: Survival of *Lactobacillus gasseri* (LG) at in vitro gastrointestinal conditions of the ALG AM, ALG UT, and cell-free systems (*n* = 2), Table S1: Viability of capsules loaded with *L. gasseri*.
