# Supplementary material for: Encapsulation of Lactobacillus gasseri: Characterization, Probiotic Survival, In Vitro Evaluation and Viability in Apple Juice
Source: Foods. 2022 Mar 2;11(5):740. doi: 10.3390/foods11050740 (PMC8909321; doi:10.3390/foods11050740)
Supplement: Supplementary file 1 [file foods-11-00740-s001.zip › foods-1578696-supplementary.pdf]

Supplementary Material

Table S1. Viability of capsules loaded with *Lactobacillus gasseri.*

Method	log CFU/mL (N_0)	log CFU/mL (N)	Viability (%)
ALG UT	7.420 ± 0.059[a]	4.877 ± 0.211[b]	65.73
ALG AM	7.372 ± 0.080[a]	4.858 ± 0.199[b]	65.90

ALG AM = Alginate with pretreatment magnetic stirring; ALG UT = Alginate with pretreatment Ultraturrax®, N_0 = initial concentration and N = final concentration. Means ± SD (p <0.05). Equal letters between columns means that there is no statistically significant difference.

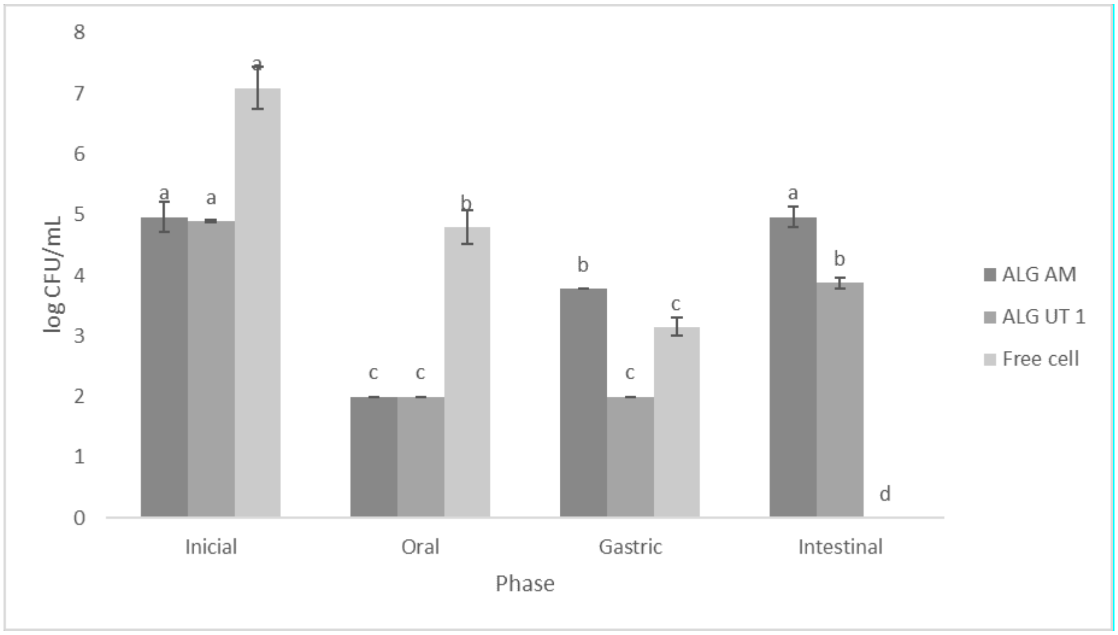

Figure S1. Survival of *Lactobacillus gasseri* at in vitro gastrointestinal conditions of the ALG AM, ALG UT, and cell-free systems (n = 2). LG AM = Alginate with pretreatment magnetic stirring; ALG UT = Alginate with pretreatment Ultraturrax®. Means ± SD (p <0.05). Equal letters means that there is no statistically significant difference.
